# Evidence for Avian Intrathoracic Air Sacs in a New Predatory Dinosaur from Argentina

**DOI:** 10.1371/journal.pone.0003303

**Published:** 2008-09-30

**Authors:** Paul C. Sereno, Ricardo N. Martinez, Jeffrey A. Wilson, David J. Varricchio, Oscar A. Alcober, Hans C. E. Larsson

**Affiliations:** 1 Department of Organismal Biology and Anatomy, University of Chicago, Chicago, Illinois, United States of America; 2 Museo de Ciencias Naturales, San Juan, Argentina; 3 Museum of Paleontology and Department of Geological Sciences, University of Michigan, Ann Arbor, Michigan, United States of America; 4 Department of Earth Sciences, Montana State University, Bozeman, Montana, United States of America; 5 Redpath Museum, McGill University, Montreal, Quebec, Canada; University of Oxford, United Kingdom

## Abstract

**Background:**

Living birds possess a unique heterogeneous pulmonary system composed of a rigid, dorsally-anchored lung and several compliant air sacs that operate as bellows, driving inspired air through the lung. Evidence from the fossil record for the origin and evolution of this system is extremely limited, because lungs do not fossilize and because the bellow-like air sacs in living birds only rarely penetrate (pneumatize) skeletal bone and thus leave a record of their presence.

**Methodology/Principal Findings:**

We describe a new predatory dinosaur from Upper Cretaceous rocks in Argentina, *Aerosteon riocoloradensis* gen. et sp. nov., that exhibits extreme pneumatization of skeletal bone, including pneumatic hollowing of the furcula and ilium. In living birds, these two bones are pneumatized by diverticulae of air sacs (clavicular, abdominal) that are involved in pulmonary ventilation. We also describe several pneumatized gastralia (“stomach ribs”), which suggest that diverticulae of the air sac system were present in surface tissues of the thorax.

**Conclusions/Significance:**

We present a four-phase model for the evolution of avian air sacs and costosternal-driven lung ventilation based on the known fossil record of theropod dinosaurs and osteological correlates in extant birds:

(1) Phase I—Elaboration of paraxial cervical air sacs in basal theropods no later than the earliest Late Triassic.

(2) Phase II—Differentiation of avian ventilatory air sacs, including both cranial (clavicular air sac) and caudal (abdominal air sac) divisions, in basal tetanurans during the Jurassic. A heterogeneous respiratory tract with compliant air sacs, in turn, suggests the presence of rigid, dorsally attached lungs with flow-through ventilation.

(3) Phase III—Evolution of a primitive costosternal pump in maniraptoriform theropods before the close of the Jurassic.

(4) Phase IV—Evolution of an advanced costosternal pump in maniraptoran theropods before the close of the Jurassic.

In addition, we conclude:

(5) The advent of avian unidirectional lung ventilation is not possible to pinpoint, as osteological correlates have yet to be identified for uni- or bidirectional lung ventilation.

(6) The origin and evolution of avian air sacs may have been driven by one or more of the following three factors: flow-through lung ventilation, locomotory balance, and/or thermal regulation.

## Introduction

**Figure 1:**
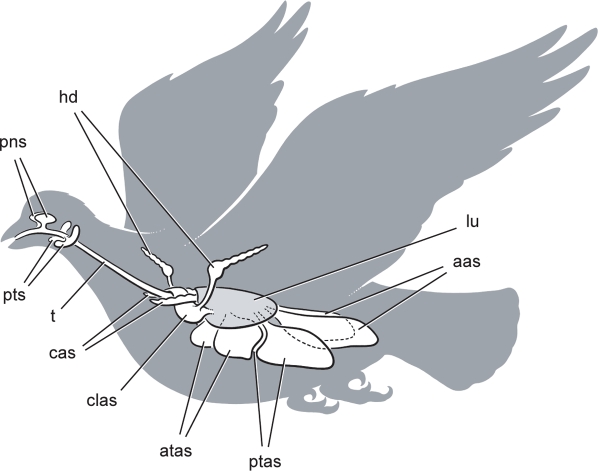
Cranial sinus and postcranial air sac systems in birds. All pneumatic spaces are paired except the clavicular air sac, and the lungs are shaded. *Abbreviations: aas*, abdominal air sac; *atas*, anterior thoracic air sac; *cas*, cervical air sac; *clas*, clavicular air sac; *hd*, humeral diverticulum of the clavicular air sac; *lu*, lung; *pns*, paranasal sinus; *ptas*, posterior thoracic air sac; *pts*, paratympanic sinus; *t*, trachea.

The respiratory tract of birds has an elaborate series of pneumatic (air-filled) outgrowths that include *sinuses* within the head and neck and *air sacs* within the thorax (Figure 1). Sinuses often invade the bones enclosing the nasal cavity (paranasal sinuses) and ear region (paratympanic sinuses), forming membrane-lined, air-filled internal spaces. Air sacs also invade bone in the postcranial skeleton, although many remain free of bone so they can function as compliant bellows, shunting inspired air along a path through a pair of rigid lungs [1]–[4].

In this paper, we describe a new large-bodied theropod from the Late Cretaceous of Argentina, *Aerosteon riocoloradensis* gen. et sp. nov., characterized by cranial and postcranial bones that are exceptionally pneumatic. Some of its postcranial bones show pneumatic hollowing that can be linked to intrathoracic air sacs that are directly involved in lung ventilation. As a result of an extraordinary level of pneumatization, as well as the excellent state of preservation of much of the axial column and girdles, *Aerosteon* helps to constrain hypotheses for the evolution of avian-style respiration.

The new theropod is particularly interesting for another reason; it represents a previously unrecorded lineage of large-bodied predator in the early Late Cretaceous (Santonian, ca. 84 Ma) of South America. Most large-bodied Cretaceous theropods on southern continents (South America, Africa, Madagascar, India) pertain to one of three distinctive and contemporary clades: abelisaurids [5]–[7], spinosaurids [8], [9], or carcharodontosaurids [10]–[12]. This predatory triumvirate persisted for millions of years on both South America and Africa [13]. Although initially thought to be a late-surviving carcharodontosaurid [14], *Aerosteon* preserves cranial bones that bear none of the distinguishing features of carcharodontosaurids [13], [15]. Rather, *Aerosteon* represents a distinctive basal tetanuran lineage that has survived into the Late Cretaceous on South America and is possibly linked to the allosauroid radiation of the Jurassic.

The purpose of the present paper is not to determine the precise phylogenetic position of *Aerosteon* but rather to describe its most salient features, outline the range of its remarkable pneumatic spaces, and discuss their relevance to understanding the early evolution of avian air sacs and lung ventilation.

### Avian Air Sacs

Avian air sacs arise directly from the lungs (Figure 1) and can be divided into *cranial* and *caudal* divisions [1]–[4], [16]–[22]. Within the cranial division, the paired *cervical* air sacs are not ventilated during respiration and often invade the centrum and neural arches of cervical and anterior thoracic vertebrae. The primary role of the cervical air sacs seems to be the reduction of bone mass, as shown by quantitative comparison of the extent of pneumatic hollowing of skeletal bone in volant and nonvolant birds [22].

The remaining air sacs are involved in lung ventilation and include the median *clavicular* air sac, the paired *anterior* and *posterior thoracic* air sacs, and the paired *abdominal* air sacs (Figure 1). In contrast to cervical air sacs, these operate as bellows, shunting inspired air along a unidirectional pathway through the lungs—a respiratory pattern unique to birds [1]–[4]. Whereas the anterior and posterior thoracic air sacs almost never invade bone in living birds, the clavicular and abdominal air sacs more commonly have diverticulae that invade axial or appendicular bone [16]–[22]. The intrathoracic location and form of these pneumatic invasions in living birds constitutes the available comparative evidence to allow their identification in fossils [23]. With the exception of evidence presented below, however, pneumatic invasion of appendicular skeletal bone within the thorax has not been reported on conclusive evidence outside crown birds (Neornithes).

### Fossil Evidence

#### Cervical Air Sacs

The cervical air sacs lie alongside the vertebral column in living birds and sometimes invade the vertebrae and ribs via pneumatopores of variable size. Invaginated pneumatic spaces that enter the lateral aspect of the centra, called *pleurocoels*, have been traced back in the fossil record to the Late Jurassic bird *Archaeopteryx* [23]–[25], to various saurischian dinosaurs [23], [26], [27], pterosaurs [23], [28], [29], and possibly to even more primitive, crocodilian relatives in the Triassic [30]. Although doubt was cast recently on the use of fossae as evidence of pneumaticity in basal archosaurs [31], a new basal suchian (distant crocodilian relative) has just been described with cervical pleurocoels [32].

For more than a century, paleontologists have agreed on the existence of cervical air sacs, or some comparable pneumatic structure, in saurischian dinosaurs on the basis of pleurocoels which closely resemble those in some living birds. The longstanding question is the fossil record of noncervical air sacs—sacs that are involved in avian lung ventilation.

#### Ventilatory Air Sacs

Ventilatory air sacs, unfortunately, are the least likely to leave evidence of their presence in skeletal bone. Paleontologists and comparative anatomists, as a result, have focused on axial pneumaticity, and opinion has split as to the meaning of observed patterns. In extant birds, pneumatic invasion by cervical air sacs is usually restricted to the cervical and anterior thoracic vertebrae and their respective ribs [1], [16]–[22]. The posterior thoracic, synsacral, and caudal vertebrae, in contrast, are pneumatized by diverticulae extending directly from the lung or from abdominal air sacs [1], [16], [19], [21], [22]. Some authors have concluded, therefore, that the lung and abdominal air sacs must also be responsible for pneumaticity in the posterior half of the axial column in nonavian dinosaurs and, on this basis, have packed the thoracic cavity of theropods with a full complement of avian ventilatory air sacs [33]. An opposing view is that the continuous series of pleurocoels observed in many nonavian dinosaurs suggests that the nonventilatory, paraxial cervical air sacs extended posteriorly along the column [26], [34].

We are inclined to support the latter, more conservative interpretation that pleurocoels in nonavian dinosaurs are a product of paraxial cervical air sacs and provide, at best, ambiguous evidence for intrathoracic ventilatory air sacs. First, pleurocoels are rare in birds, and no living bird has an unbroken cervical-to-caudal series of pleurocoels as occurs in some nonavian dinosaurs, including the one we describe below [31]. As Wedel [26] has underscored, pleurocoels extend posteriorly in the axial column of saurischian dinosaurs to a variable extent, but neither adults nor juveniles of any species show an apneumatic gap. Allotting an unbroken series of pleurocoels of graded form, as in the case we describe below, to three different pneumatic sources (cervical air sacs, lung diverticulae, abdominal air sacs) is difficult to defend. Drawing a direct analogy based on birds for the source(s) of pneumaticity in the posterior axial column in nonavian dinosaurs [22], [31], [33], thus, is problematic.

Second, cervical air sacs have been observed extending to the posterior end of the vertebral column in birds. Several authors have described cervical air sacs extending posteriorly beyond the abdominal air sacs in the ostrich (*Struthio camelus*) [21], [36]. Ratites have relatively smaller abdominal sacs than in other birds and, as nonvolant basal avians, serve as better analogs for nonavian saurischians than volant neognaths [37].

Finally, the posterior portion of the avian axial skeleton is completely transformed by extensive coossification of vertebrae and girdle bone and by posterolateral rotation of the pubes away from the midline, which allows unobstructed distal extension of the viscera and abdominal air sac under the synsacrum and caudal vertebrae. The pelvic space in nonavian saurischians, in contrast, is not nearly as open and continuous with the thoracic cavity, offering comparably less space for these air sacs [37].

More conclusive evidence of intrathoracic ventilatory air sacs in fossils, in sum, will require an osteological signature of pneumatic structures in nonaxial (appendicular) bones, which that cannot be dismissed as an elaboration of the paraxial cervical air sacs. We now turn attention to previous reports of such pneumaticity in the appendicular skeleton of nonavian dinosaurs.

#### Previous Reports of Appendicular Pneumaticity

Appendicular pneumaticity in nonavian dinosaurs is poorly documented. An ilium of the Middle Jurassic theropod *Piatnitzkysaurus* was shown with two small foramina, one above the pubic peduncle and another above the acetabulum [38: fig. 22]. These small foramina were described as pneumatic and associated with internal space and likened to other supposedly pneumatic foramina in the femur, tibia and metatarsals. The small foramen figured on the shaft of the tibia has elsewhere been described as a neurovascular foramen. Were such long bones also pneumatic, this would constitute an extraordinary condition, as none of the limb bones in *Archaeopteryx* nor those in other basal avians outside Ornithothoraces (Enantiornithes + Euornithes) has ever been shown to be pneumatic.

A deep depression on the brevis shelf on an ilium of the Cretaceous carcharodontosaurid theropod *Mapusaurus*, likewise, was labeled “pneumatic diverticulae” of the ilium of [12: fig 26]. The structure, however, was described in the text as a site of origin of powerful hind limb retractor musculature. Whether the ilium in *Mapusaurus* is actually pneumatized, however, cannot be determined on available information.

Recently, the furcula in the Early Cretaceous dromaeosaurid *Buitreraptor* was described as “pneumatic” with “trabeculae spanning its interior” [39:1008]. Most of the interior of the bone is hollowed, which does suggest pneumatic invasion, although the pair of potential pneumatopores near the midline are particularly small (P. Makovicky, pers. comm.).

“Cancellous” bone was described in the ilia of the titanosaurs *Epachthosaurus* [40] and *Lirainosaurus* [41], although no further claim was made regarding its pneumatic status. The titanosauriform *Euhelopus* also has a bone texture of larger cells that may be pneumatic (J. Wilson, personal observation). At present there are no reports of appendicular bones among sauropodomorphs with pneumatopores that lead to bone texture than is demonstrably pneumatic.

In sum, the evidence currently documenting the presence of nonaxial pneumatic structures in nonavian dinosaurs is poorly established. Several years ago, we reported the pneumatic spaces in the furcula and ilium described below [14]. The pneumatic status of these and other structures we describe below is quite evident and will add important new evidence for the identity and distribution of air sacs in nonavian theropods.

## Methods

### Preparation

The holotypic and referred specimens were prepared using pin vice, pneumatic air scribe, and air abrasive. The white-colored bones are embedded in a fine-grained, poorly sorted, hematitic siltstone matrix that in places was very hard. To reduce color distractions in photographic images between the white bone and red-brown matrix, some bones were molded and cast in matt grey-colored casting epoxy.

### Imaging

Computed tomography of the furcula, gastral elements, and ilium was undertaken to observe internal pneumatic spaces using a Philips Brilliance 64-slice scanner at 80 Kv in the University of Chicago Hospitals.

### Terminology

We employed traditional, or “Romerian,” anatomical and directional terms over veterinarian alternatives [42] and followed recent recommendations regarding the identification of vertebral laminae [43]. “Anterior” and “posterior,” for example, are used as directional terms rather than the veterinarian alternatives “rostral” or “cranial” and “caudal,” except when referring to cranial and caudal air sac divisions in birds.

**Institutional abbreviations:**

**Table:** 

FMNH	Field Museum of Natural History, Chicago, Illinois, United States of America.
LH	Las Hoyas collection, Museo de Cuenca, Cuenca, Spain.
MCNA	Museo de Ciencias Naturales y Antropológicas (J. C. Moyano) de Mendoza, Mendoza, Argentina.
MNN	Muséum National du Niger, Niamey, République de Niger.

## Results

### Systematic Paleontology

**Systematic hierarchy:**

Dinosauria Owen, 1842

Theropoda Marsh, 1881

Tetanurae Gauthier, 1986

Allosauroidea Marsh, 1878

***Aerosteon*** **gen. nov.**

#### Etymology

*Aeros*, air (Greek); *osteon*, bone (Greek). Named for the extreme development of pneumatic spaces in skeletal bone.

#### Type Species

*Aerosteon riocoloradensis.*

***Aerosteon riocoloradensis*** **sp. nov.**

**Figure 2:**
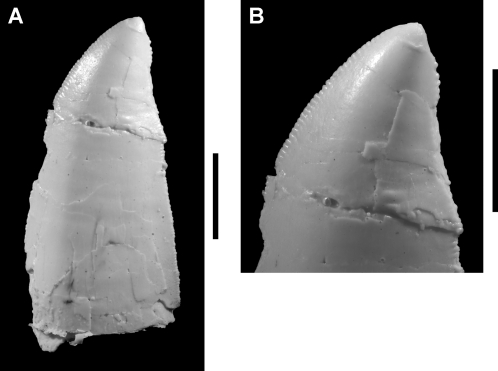
Tooth of the theropod *Aerosteon riocoloradensis*. Isolated crown from the maxillary or dentary series (MCNA-PV-3137; cast). (A)-Side view of crown. (B)-Enlarged view of crown tip. Scale bars equal 1 cm.

Figures 2–11, 12A, 13–16

**Figure 3 pone-0003303-g003:**
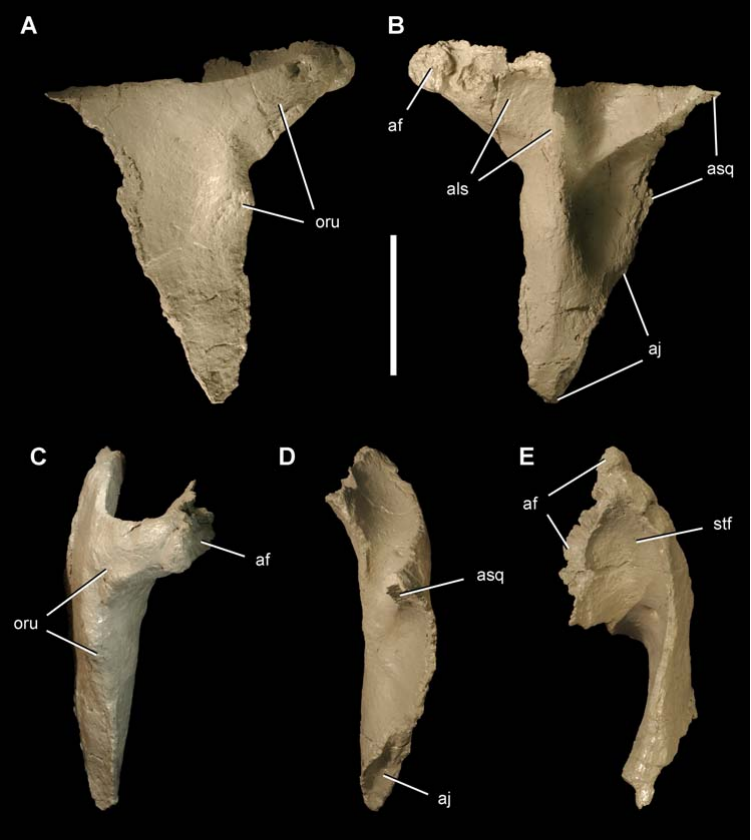
Postorbital of the theropod *Aerosteon riocoloradensis*. Right postorbital (MCNA-PV-3137; cast) in right lateral (A), medial (B), anterior (C), posterior (D), and dorsal (E) views. Scale bar equals 5 cm. *Abbreviations*: *af*, articular surface for the frontal; *aj*, articular surface for the jugal; *als*, articular surface for the laterosphenoid; *asq*, articular surface for the squamosal; *oru*, orbital rugosity; *stf*, supratemporal fossa.

**Figure 4 pone-0003303-g004:**
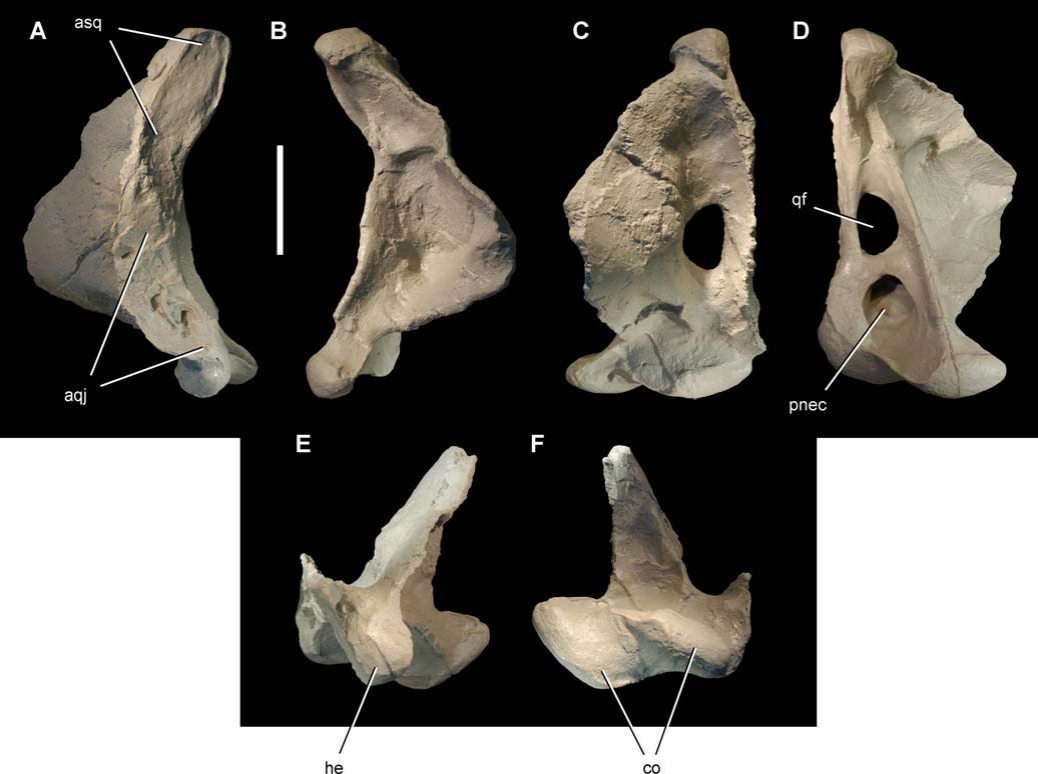
Quadrate of the theropod *Aerosteon riocoloradensis*. Left quadrate (MCNA-PV-3137; cast) in left lateral (A), medial (B), anterior (C), posterior (D), dorsal (E), and ventral (F) views. Scale bar equals 5 cm. *Abbreviations*: *aqj*, articular surface for the quadratojugal; *asq*, articular surface for the squamosal; *co*, condyle; *he*, head; *pnec*, pneumatocoel; *qf*, quadrate foramen.

**Figure 5 pone-0003303-g005:**
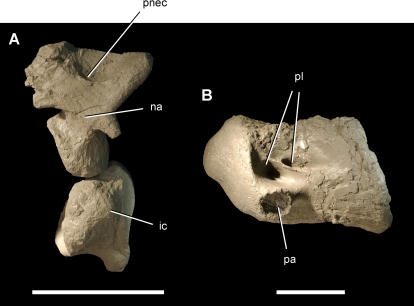
Anterior cervical vertebrae of the theropod *Aerosteon riocoloradensis*. Atlas and cervical 3 centrum (MCNA-PV-3137; cast) in left lateral view. (A)-Atlas. (B)-Cervical 3 centrum. Scale bars equal 5 cm. *Abbreviations*: *ic*, intercentrum; *na*, neural arch; *pa*, parapophysis; *pl*, pleurocoel; *pnec*, pneumatocoel.

**Figure 6 pone-0003303-g006:**
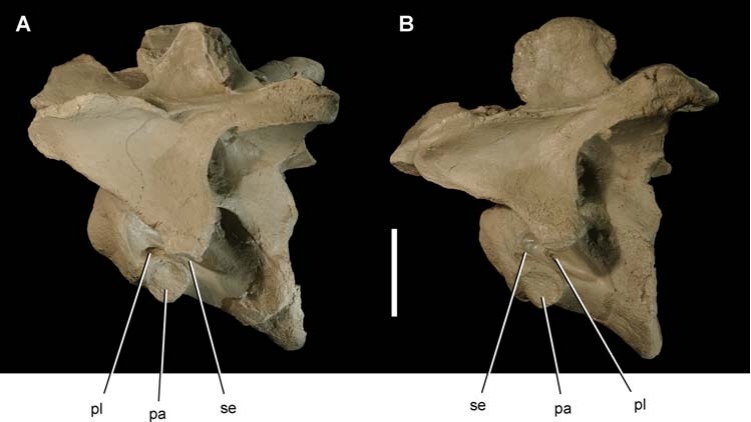
Mid cervical vertebrae of the theropod *Aerosteon riocoloradensis*. Cervical vertebrae 4 and 6 (MCNA-PV-3137; cast) in left lateral view. (A)-Cervical 4. (B)-Cervical 6. Scale bar equals 5 cm. *Abbreviations*: *pa*, parapophysis; *pl*, pleurocoel; *se*, septum.

**Figure 7 pone-0003303-g007:**
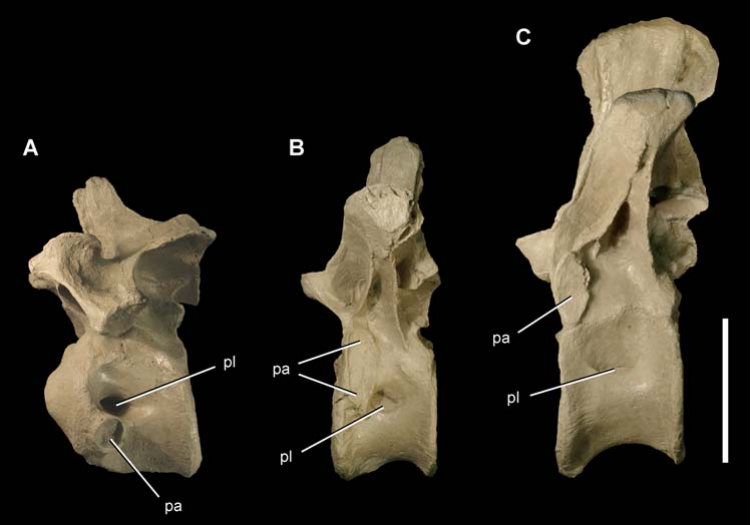
Anterior and mid dorsal vertebrae of the theropod *Aerosteon riocoloradensis*. Dorsal vertebrae 1, 4 and 8 (MCNA-PV-3137; cast) in left lateral view. (A)-Dorsal 1. (B)-Dorsal 4. (C)-Dorsal 8. Scale bar equals 10 cm. *Abbreviations*: *pa*, parapophysis; *pl*, pleurocoel.

**Figure 8 pone-0003303-g008:**
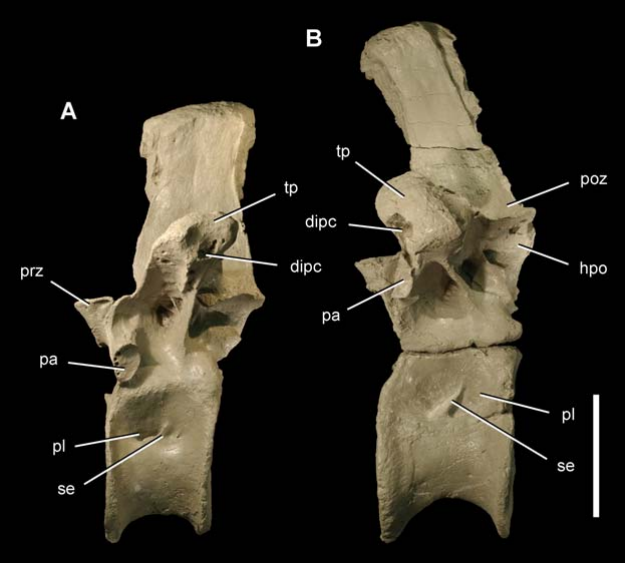
Posterior dorsal vertebrae of the theropod *Aerosteon riocoloradensis*. Dorsal vertebrae 11 and 14 (MCNA-PV-3137; cast) in left lateral view. (A)-Dorsal 11. (B)-Dorsal 14. Scale bar equals 10 cm. *Abbreviations*: *dipc*, diapophyseal canal; *hpo*, hyposphene; *pa*, parapophysis; *pl*, pleurocoel; *poz*, postzygapophysis; *prz*, prezygapophysis; *se*, septum; *tp*, transverse process.

**Figure 9 pone-0003303-g009:**
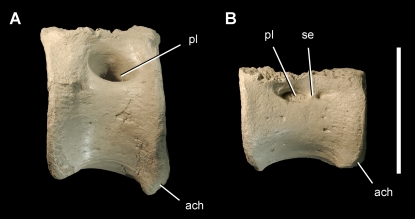
Caudal vertebrae of the theropod *Aerosteon riocoloradensis*. Anterior and mid caudal centra (MCNA-PV-3137; cast) in left lateral view. (A)-Anterior caudal centrum. (B)-Mid caudal centrum. Scale bar equals 10 cm. *Abbreviations*: *ach*, articular surface for a chevron; *pl*, pleurocoel; *se*, septum.

**Figure 10 pone-0003303-g010:**
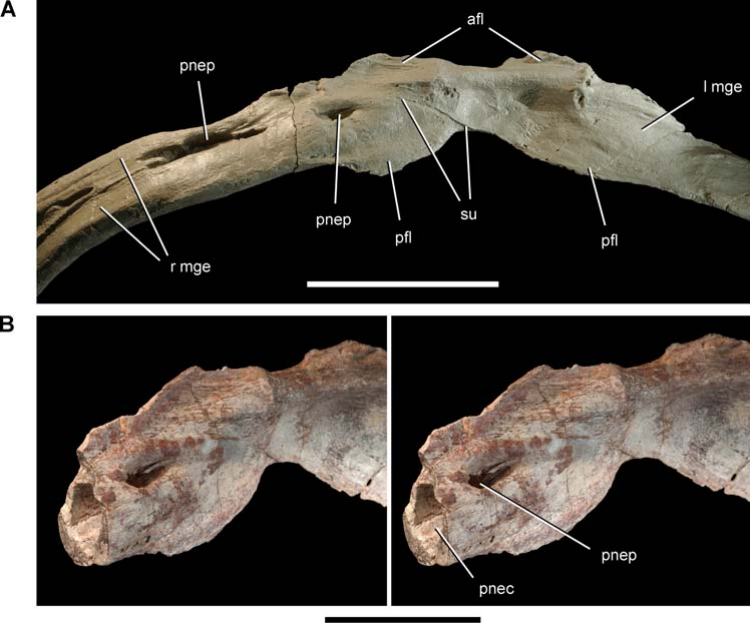
Gastralia of the theropod *Aerosteon riocoloradensis.* Coossified medial gastral elements from anterior end of cuirass (MCNA-PV-3137). (A)-Coossified gastralia (cast) in ventral view. (B)-Stereopairs of the medial portion of one gastralium showing the pneumatopore and lumen inside the shaft in ventrolateral view. Scale bars equal 10 cm in A and 5 cm in B. *Abbreviations*: *afl*, anterior flange; *l*, left; *mge*, medial gastral element; *pfl*, posterior flange; *pnec*, pneumatocoel; *pnep*, pneumatopore; *r*, right; *su*, suture.

**Figure 11:**
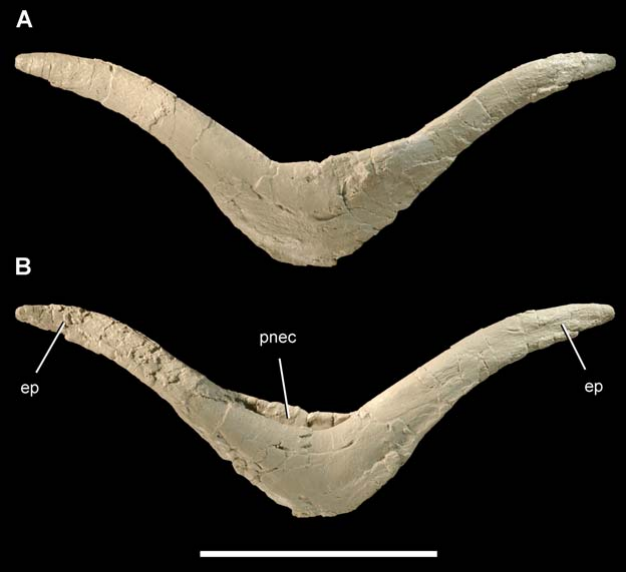
Furcula of the theropod *Aerosteon riocoloradensis.* Furcula (MCNA-PV-3137; cast) in anterior (A) and posterior (B) views. Scale bar equals 10 cm. *Abbreviations*: *ep*, epicleideum; *pnec*, pneumatocoel.

**Figure 12 pone-0003303-g012:**
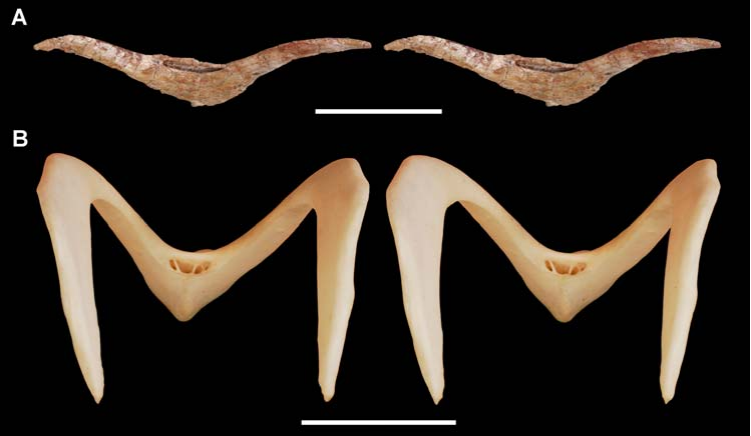
Furcula of the theropod *Aerosteon riocoloradensis* and magpie goose *Anseranas semiplamata.* (A)-Stereopairs of the furcula of *Aerosteon riocoloradensis* (MCNA-PV-3137) in posterodorsal view. (B)-Stereopairs of the furcula of *Anseranas semiplamata* (FMNH 338808) in posterodorsal view. Scale bars equal 10 cm in A and 2 cm in B.

**Figure 13 pone-0003303-g013:**
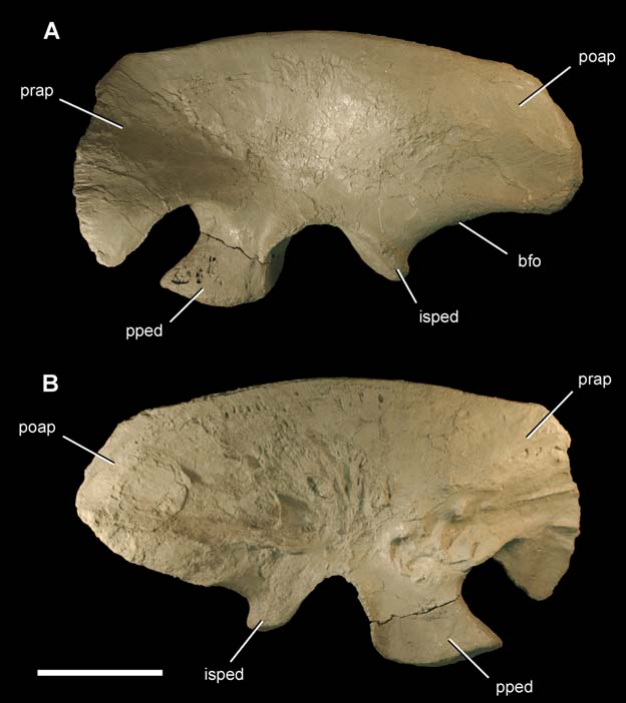
Ilium of the theropod *Aerosteon riocoloradensis.* Left ilium (MCNA-PV-3137; cast) in left lateral (A) and medial (B) views. Scale bar equals 20 cm. *Abbreviations*: *bfo*, brevis fossa; *isped*, ischial peduncle; *poap*, postacetabular process; *pped*, pubic peduncle; *prap*, preacetabular process.

**Figure 14 pone-0003303-g014:**
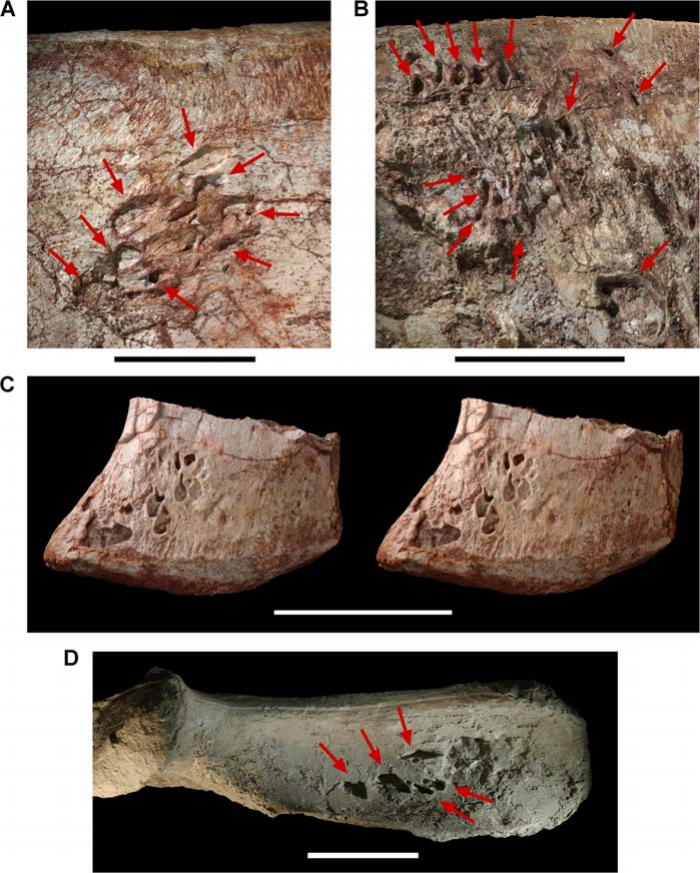
Pneumatopores on the left ilium of the theropod *Aerosteon riocoloradensis.* Detail views of the left ilium (MCNA-PV-3137). (A)-Pneumatopores on the base of the preacetabular process in lateral view. (B)-Pneumatopores on the central iliac blade in medial view. (C)-Stereopairs of the pubic peduncle in lateral view showing pneumatopore complex. (D)-Pneumatopores on the brevis fossa of the postacetabular process in ventral view; largest pneumatopore (4 cm in transverse diameter) opens posteriorly (to the right) just posterior to five smaller ventrally-facing pneumatopores (marked). A, B, and D are from a cast of MCNA-PV-3137 to reduce color distraction. Scale bars equal 5 cm in A and 10 cm in B, C and D. Arrows point to pneumatopores.

**Figure 15 pone-0003303-g015:**
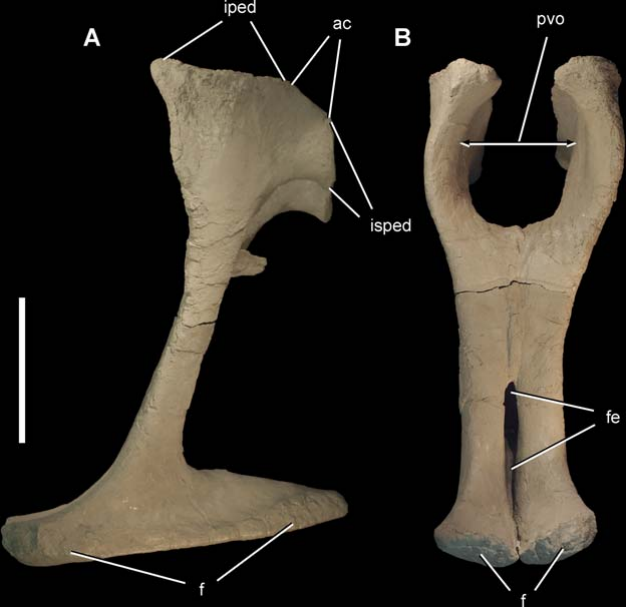
Pubes of the theropod *Aerosteon riocoloradensis.* Pubes (MCNA-PV-3137; cast) in left lateral (A) and anterior (B) views. Scale bar equals 20 cm. *Abbreviations*: *ac,* acetabulum; *f*, foot; *fe*, fenestra; *iped*, iliac peduncle; *isped*, ischial peduncle; *pvo*, pelvic outlet.

**Figure 16:**
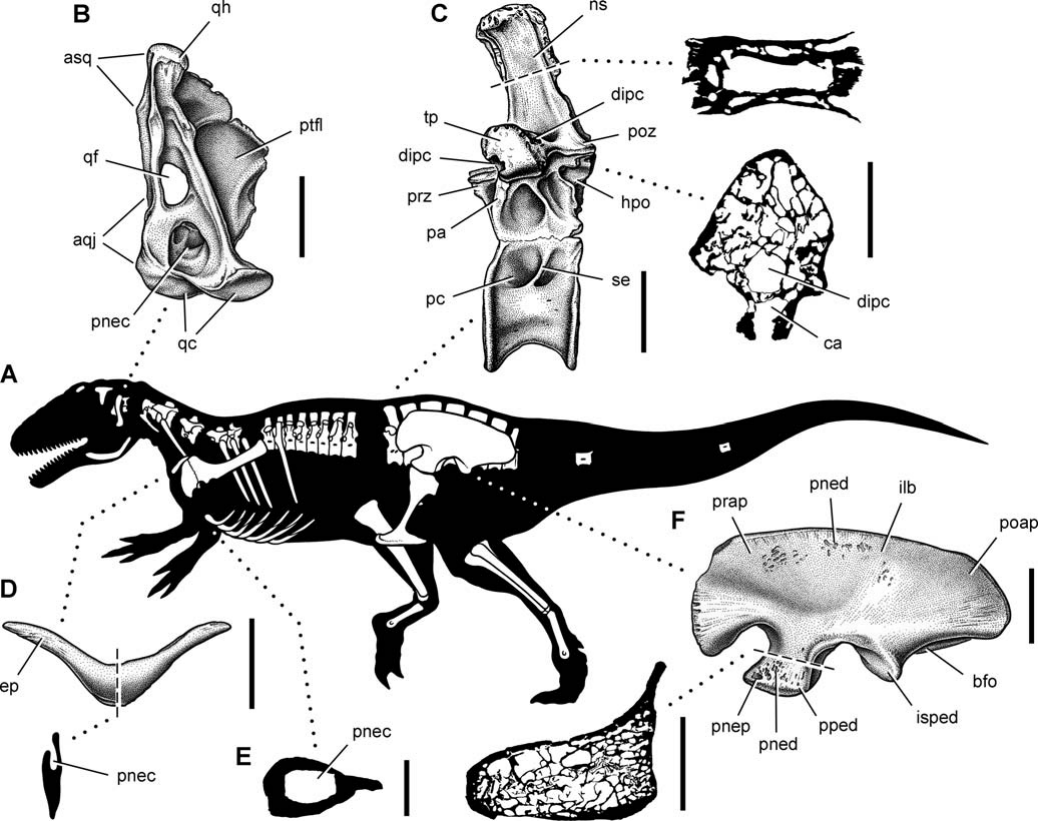
Summary of pneumatic features of the theropod *Aerosteon riocoloradensis*. (A)-Silhouette reconstruction in left lateral view showing preserved bones of the holotype and referred specimens (MCNA-PV-3137-3139); body length approximately 9-10 m. (B)-Left quadrate in posterior view. (C)-Dorsal 14 in left lateral view with enlarged cross-sections of the neural spine and transverse process. (D)-Furcula in anterior view with sagittal cross-section. (E)-Cross-section of medial gastral element from the anterior end of the cuirass showing pneumatocoel. (F)-Left ilium in lateral view with enlarged cross-section of pubic peduncle. Scale bars equal 5 cm in B, 10 cm (3 cm for cross-sections) in C, 10 cm (same for cross-section) in D, 2 cm in E, and 20 cm (6 cm for cross-section) in F. *Abbreviations: aqj*, articular surface for the quadratojugal; *asq*, articular surface for the squamosal; *bfo*, brevis fossa; *ca*, canal; *dipc*, diapophyseal canal; *ep*, epicleideum; *hpo*, hyposphene; *ilb*, iliac blade; *isped*, ischial peduncle; *ns*, neural spine; *pa*, parapophysis; *pc*, pleurocoel; *pnec*, pneumatocoel; *pned*, pneumatic depression; *pnep*, pneumatopore; *poap*, postacetabular process; *poz*, postzygapophysis; *pped*, pubic peduncle; *prap*, preacetabular process; *prz*, prezygapophysis; *ptfl*, pterygoid flange; *qc*, quadrate condyles; *qf*, quadrate foramen; *qh*, quadrate head; *se*, septum; *tp*, transverse process.

#### Etymology

*riocolorado*, Río Colorado (Spanish); *ensis*, place (Latin). Named for the site of discovery of the holotype.

#### Holotype

MCNA-PV-3137; one maxillary or dentary crown, left prefrontal, right postorbital, left quadrate, posterior portion of the left pterygoid, right prearticular, partial or complete vertebrae including C1, C3, C4, C6, C8, D1, D4–11, D14, S2–5, CA1, one mid and distal caudal centrum, several cervical and dorsal ribs, gastralia, furcula, left scapulocoracoid, left ilium, and right and left pubes. The bones were found disarticulated but in close association over an area of 11 m^2^. The specimen is an immature individual with many open neurocentral sutures. The specimen, however, may have been approaching maturation, as indicated by fusion of the centrum and neural arch of several vertebrae and fusion of the coracoid and scapula. Body length is approximately 9–10 m, or roughly equivalent to the larger body size range for *Allosaurus*.

#### Type Locality

Cañadon Amarillo (S 37.5°, W 70.5°), north of Cerro Colorado, 1 km north of the Río Colorado near the southern border of Mendoza Province, Argentina.

#### Horizon

Anacleto Formation, Neuquén Group; Santonian (ca. 84 Mya) [44], [45]. Titanosaurian sauropods are common in the formation.

#### Diagnosis

*Aerosteon riocoloradensis* is characterized by a prefrontal with a very short ventral process, an enlarged quadrate foramen located entirely within the quadrate, a large tympanic diverticulum into the quadrate shaft above the articular condyle, anterior dorsal vertebra with very large parapophyses, dorsal neural spines with central pneumatic space, posteriormost dorsal vertebra with anterodorsally inclined neural spine and a pneumatic canal within the transverse process, anterior caudal vertebrae with a large pleurocoel, medial gastral elements coossified with anterior and posterior flanges, and a furcula with median pneumatocoel.

These features distinguish *Aerosteon riocoloradensis* from known South American abelisaurids, as represented by *Carnotaurus sastrei* [5] and *Illokelesia aguadagrandensis* [6], spinosaurids such as *Irritator challengeri* [9], and carcharodontosaurids such as *Giganotosaurus carolinii* [10], *Mapusaurus rosae* [12] or *Tyrannotitan chubutensis* [46].

#### Referred Material

MCNA-PV-3138, left metatarsal 2; MCNA-PV-3139, articulated left tibia, fibula, astragalus and calcaneum lacking only the proximal end of the fibula. This material is tentatively referred to *A. coloradensis* on the basis of its horizon and morphology, the latter impossible to overlap with the holotypic skeleton but consistent as pertaining to a basal tetanuran. Both referred specimens pertain to slightly smaller individuals that the holotype. MCNA-PV-3138 was found approximately 10 m away from the holotypic locality. MCNA-PV-3139 was found several kilometers distant.

### Description

We briefly comment below on the morphology of *Aerosteon riocoloradensis* and provide some basic cranial and postcranial measurements (Tables 1–3). An isolated crown of relatively small size was found at the site and has approximately three serrations per millimeter on both mesial and distal margins (Figure 2).

**Table 1:** Dimensions (cm) of isolated crown and select cranial bones of the holotypic specimen of *Aerosteon riocoloradensis* (MCNA-PV-3137).

Bone	Measurement	Length
Crown	Height Base, mesiodistal length Base, maximum labiolingual width	3.8 1.7 1.0
Prefrontal	Maximum length Maximum width	6.8 2.3
Postorbital	Maximum height Maximum anteroposterior length	11.5 11.4
Quadrate	Maximum height Head, anteroposterior length Head, transverse width Quadrate foramen, maximum height Quadrate foramen, maximum width Transverse width across condyles	16.3 2.6 2.4 2.7 1.7 7.9

**Table 2 pone-0003303-t002:** Dimensions (cm) of select vertebrae (Figures 5–9) from the holotypic specimen of *Aerosteon riocoloradensis* (MCNA-PV-3137).

Vertebra	Centrum Length^1^	Posterior Centrum Height	Posterior Centrum Width	Pleurocoel Morphology
C1	2.5^2^	3.3^2^	6.5^2^	—3
C3	9.6	8.4	9.2	Two; dorsal smaller, septum inclined anterodorsally
C4	9.8	8.4	9.2	Two; subequal, septum inclined anterodorsally
C6	9.1	8.1	8.4	Two; dorsal is rudimentary, septum inclined anterodorsally
D1	8.5	8.8	9.4	One; oval, large
D4	7.1	8.7	8.8	One; oval, medium-sized
D8	8.8	10.8	9.8	One; oval, medium-sized
D10	8.4	13.1	11.3	One; oval, medium-sized
D11	8.4	12.1	11.1	Two; posterior is rudimentary, septum inclined posterodorsally
D14	10.2	15.0	13.5	Two; subequal in size, septum inclined posterodorsally
CA1	9.3	11.8	12.8	One; large with recessed septae
mid CA	10.0	7.7	7.7	Two; posterior is rudimentary^4^

*Abbreviations*: C, cervical; CA, caudal; D, dorsal.

1 Measured along ventral edge excluding anterior convexity of centrum when present.

2 Measurement pertains to intercentrum.

3 Intercentrum does not appear to be pneumatic; neural arch has single lateral pneumatocoel.

4 Right side has only one pleurocoel (left side shown in Figure 9B).

**Table 3:** Dimensions (cm) of girdle bones (Figures 11–15) of the holotypic specimen of *Aerosteon riocoloradensis* (MCNA-PV-3137).

Bone	Measurement	Length, Angle (cm, degrees)
Scapulocoracoid	Coracoid, maximum height Posterior process (glenoid to tip of process) Scapular length Scapular blade, minimum width Scapular blade, distal width Height (glenoid to acromion) Acromion width Glenoid, maximum length	27.6 10.0 57.0 7.6 11.0 21.0 9.5 8.7
Furcula	Maximum transverse width Intrafurcular angle	25.2 120°
Ilium	Iliac blade, maximum length Iliac blade, height above acetabulum Preacetabular process length Preacetabular process, height at base Preacetabular process, height at distal end Postacetabular process length Postacetabular process, height at mid length Pubic peduncle, anteroposterior length Pubic peduncle, maximum transverse width Acetabulum, maximum width	76.8 30.0 16.8 24.4 33.5 28.5 25.0 16.9 8.1 10.3
Pubis	Length (acetabulum to foot) Iliac peduncle length Ischial peduncle length Pubic foot, maximum length Pubic foot, maximum width	62.0 18.0 9.0 47.2 19.3
Pelvic outlet (between pubes)	Maximum width Minimum width between ischial peduncles Minimum width between iliac peduncles	14.0 11.2 7.0

#### Cranium

Of the preserved cranial bones (prefrontal, postorbital, quadrate, posterior pterygoid part, prearticular), the prefrontal, postorbital and quadrate are the most informative. The prefrontal is complete and lacks a long ventral process along the orbital margin, unlike most basal tetanurans such as *Allosaurus* [47] and *Sinraptor* [48].

The triradiate postorbital has a short posterior process, a subtriangular ventral process, and a blunt medial process (Figure 3). In lateral view, the orbit margin is only slightly raised and rugose (Figure 3A). It does not exhibit the expanded brow ridge common to *Acrocanthosaurus* [49], [50] and other carcharodontosaurids [11], [15], and the medial socket for the head of the laterosphenoid is particularly shallow (Figure 3B), unlike either abelisaurids [51] or carcharodontosaurids [15].

The quadrate (Figures 4, 16B) has an enlarged fenestra bounded entirely by the shaft rather than shared by the quadrate and quadratojugal as in many other theropods [47], [48]. Just below that fenestra is a large pneumatocoel that extends as a blind fossa deep within the quadrate at the base of the pterygoid process. The fossa descends to the edge of the quadrate condyles, where it would likely have crossed the jaw joint and entered the lower jaw, as does the paratympanic sinus in many living birds and crocodilians [52]. This pneumatic fossa is much better developed than depressions on the quadrate shaft in the carcharodontosaurid *Giganotosaurus* [10] or the abelisaurid *Majungasaurus* [51]. The distal condyles are very broad relative to the height of the quadrate (Table 1), which contrasts strongly with the condition in abelisaurids [51].

#### Axial Skeleton

All of the vertebrae exhibit camellate (honeycomb) internal structure in both the centrum and neural arch. The expression of a “hyperpneumatic” condition in the vertebrae is matched elsewhere by the marked pneumatic invasion of the quadrate, appendicular elements and gastralia. The heightened state of pneumatic invasion across these skeletal divisions suggests genetic control of the degree or general extent of pneumaticity [31], [52]. Such extensive camellate pneumaticity along the entire vertebral column has been reported previously in advanced coelurosaurs, such as *Tyrannosaurus* [23], [53], and in titanosaurs [23], [26], [27].

The atlas has a single slit-shaped pneumatopore in the center of the neural arch that opens dorsally (Figure 5A). The atlantal intercentrum does not appear to be pneumatic. Posterior to the atlas, the pleurocoels are paired in anterior cervical vertebrae with an anterodorsally inclined septum (Figure 5B). They grade into a single opening in the mid cervical and anterior dorsal vertebrae (Figures 6, 7), and then grade again into paired openings with an posterodorsally inclined septum in the posteriormost dorsal and sacral vertebrae (Figures 8, 16C) (Table 2).

The pneumatic features in dorsal 14 are extreme. The neural spine has a large central lumen (Figure 16C). Another lumen passes along the length of the transverse process, opening at its distal end into the rib (Figure 16C). Extensive intervertebral pneumatic communication exists via openings near the vertebral canal, between pre- and postzygapophyses, and between neural spines.

There appear to be only five sacral vertebrae in *Aerosteon* as is the case in *Allosaurus* and many other theropods outside Coelurosauria. Only fragments of sacral 1 are preserved. The middle three sacrals (S2–4) are the most complete, their centra coossified. Sacral 5 is represented by a partial centrum and transverse process. The sacral vertebrae were found together in sequence but were truncated by erosion.

Three caudal vertebrae are preserved including caudal 1, a mid caudal centrum, and a distal caudal centrum (Figure 9). A single opening is present in all vertebrae except the left side of the mid caudal, which has a second small posterior pneumatopore (Figure 9B). The internal structure of all caudal centra is camellate, as is the neural arch of caudal 1. The first caudal centrum has a large pleurocoel and a posteriorly deflected transverse process. Chevron facets are present along its posterior edge, whereas there is no indication of chevron facets anteriorly. Mid and distal caudal centra have short proportions and do not seem to exhibit the lengthening characteristic of tetanuran theropods (Figures 9B, 16A).

Cervical and dorsal ribs have pneumatic fossae or pneumatocoels opening near the junction of the capitulum and tuberculum on the anterior side of the rib head. The most extreme condition occurs in the last dorsal vertebra, in which a pneumatic canal extends within the transverse process to the rib (Figure 16C).

Gastralia are composed of medial and lateral elements. The suture between the medial and lateral elements is sinuous and long and in many cases is fused. Medial elements, with rare exception, fuse in the midline (Figure 10). Isolated medial elements show a complex articulation with the contralateral medial element in the midline, where each element is expanded with anterior and posterior flanges. Successive rows of medial elements may have been in contact along their edges, however the usual criss-cross relationship between gastral rows in the midline does not appear to have been developed. Instead, coossification of medial elements and fusion of medial and lateral elements results in single, U-shaped units composed of an opposing pair of medial and lateral elements. In one case asymmetrical fusion has welded together two successive medial elements (Figure 10A). The gastral cuirass in *Aerosteon*, as a result, seems much less flexible than in many other theropods.

Several pneumatopores open from the external side of the gastralia into the body of the element, passing internally along the gastral shaft without septae. In one case, an oval pneumatopore opens into a space within the gastralium approximately 1 cm in diameter (Figures 10B, 16E). This pneumatic excavation enters and hollows the shaft of one of the medial elements (Figure 10B). Pneumaticity within gastralia, which form in dermal bone, has never been reported previously.

#### Appendicular Skeleton

The scapulocoracoid has a subrectangular blade comparable in proportions to that in many allosauroids, such as *Sinraptor* [48]. The junction between scapula and coracoid is particularly thick, measuring more than 6 cm. The coracoid is hook-shaped.

The furcula (wishbone) is V-shaped with a broad intrafurcular angle of 120° and epicleideal processes that arch slightly to each side (Table 3). The bone is nearly flat in anterior and posterior views (Figure 11). The slit-shaped pneumatopore opens dorsally on the posterior side of the bone and has rounded margins (Figures 11B, 12A, 16D). The undivided pneumatocoel has a crescentic shape, which extends into the proximal one-half of each epicleideal process.

The ilium has a deep preacetabular process, a tapering postacetabular process, and an arched brevis fossa (Figures 13,14). The preacetabular process is weakly divided along its anterior margin into two lobes (Figure 13A). There are many small pneumatopores on each side of the blade (Figure 14). The largest of these occurs on the medial side between sacral attachment sites (Figure 14B). The pubic peduncle is robust, measuring about twice as long as broad at its distal end (Figures 13, 16F; Table 3). Several smaller pneumatocoels are grouped together on the lateral aspect of the pubic peduncle and vary in diameter from several millimeters to several centimeters (Figures 14C, 16F). The rims of these openings are finished, and all internal canals have smooth surfaces that are indisputably pneumatic in origin and communicate with the honeycomb texture within the bone (Figure 16F, enlarged cross-section). Another large pneumatic opening is located at mid length along the brevis fossa and opens posteriorly. Just proximal to this opening are five additional irregular pneumatopores that open ventrally (Figure 14D).

The articulated pubes, in contrast, do not show any external pneumatopores and are composed of dense or cancellous bone (Figure 15). A break at mid length along the pubic shafts shows they are composed of solid bone without any cancellous structure. The iliac peduncle is massive, and like the ischial peduncle is gently concave; there is no indication of a peg-in-socket articulation as occurs in some other theropods. The pubis bears a very large distal boot that is coossified with its opposite in the midline (Figure 15A). The boot measures approximately 70% the length of the pubis, which is proportionately among the largest known among theropods (Table 3).

The pelvic outlet is rather narrow dorsoventrally and transversely as in *Alllosaurus* (Figure 15B). The diameter of the posteriormost dorsal (Table 2) equals the space between the pubes, which leaves only a narrow passage for communication from the abdominal cavity to areas under the sacrum.

## Discussion

### Phylogenetic Position

**Figure 17:**
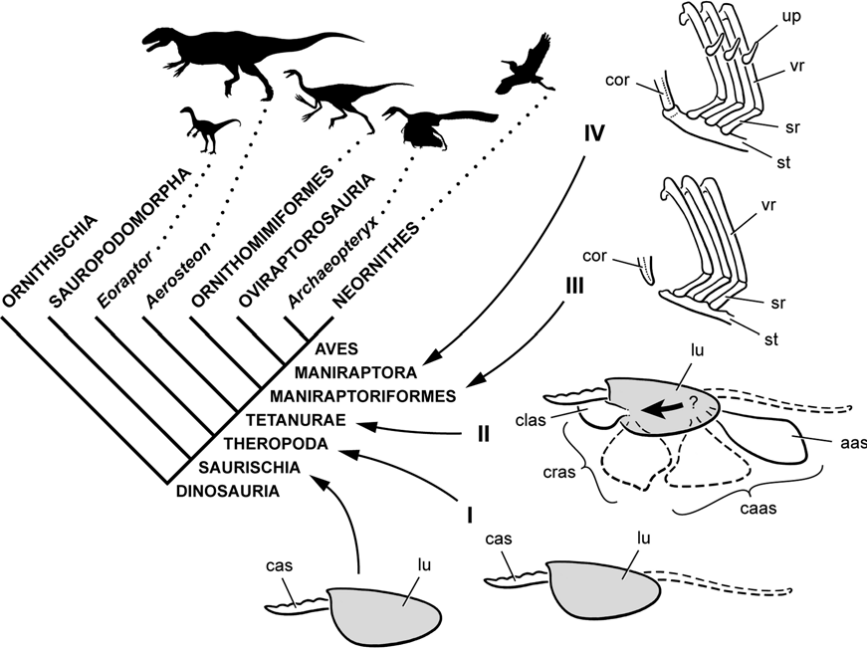
Cladogram of Dinosauria showing the four-phase model for evolution of avian air sacs and lung ventilation within Theropoda. *Phase I* (Theropoda), variable posterior extension of paraxial cervical air sacs. *Phase II* (Tetanurae), elaboration of cranial (clavicular) and caudal (abdominal) intrathoracic air sac divisions and subcutaneous diverticulae. *Phase III* (Maniraptoriformes), primitive costosternal ventilatory pump. *Phase IV* (Maniraptora), advanced costosternal ventilatory pump. *Abbreviations: aas*, abdominal air sac; *cas*, cervical air sac; *caas*, caudal air sacs; *clas*, clavicular air sac; *cor*, coracoid; *cras*, cranial air sacs; *lu*, lung; *sr*, sternal rib; *st*, sternum; *up*, uncinate process; *vr*, vertebral rib. Bold arrow on lung indicates flow-through lung ventilation; question mark indicates uncertainty in the direction of air flow (uni- or bidirectional).

The form of the pectoral and pelvic girdles in the holotypic skeleton clearly indicate that *Aerosteon* is a basal tetanuran (Figure 17). These features, which include the unexpanded crescentic coracoid, relatively broad scapular blade, relatively narrow brevis fossa and robust pubic peduncle of the ilium, and open obturator notch and large boot of the pubis, and sacrum limited to five vertebrae, closely resemble the condition in the allosauroids *Allosaurus* [47] and *Acrocanthosaurus* [49], [50], [54]. The small prefrontal and triradiate postorbital, which shows no development of a swollen brow or infraorbital flange, are closest to that in *Allosaurus* and very different from that in abelisaurids and carcharodontosaurids [15], [51].

Placing *Areosteon* more precisely among basal tetanurans is not possible without further analysis. The foregoing suggests, however, that *Areosteon* represents a new and distinctive basal tetanuran lineage recorded for the first time in Upper Cretaceous rocks in South America.

### Source of Postcranial Pneumaticity

#### Axial Pneumaticity

Inferences about postcranial pneumaticity in *Aerosteon* are very dependent on the condition in extant birds (Neornithes), the only extant outgroup with comparable skeletal structures related to pneumatization. Because only a single outgroup is available, soft-tissue inferences are weaker [55: level–two inference]. Osteological correlates must establish a convincing link for soft-tissue inference.

Anterior axial pneumaticity (cervical to anterior dorsal vertebrae plus their associated ribs) has long been attributed to cervical air sacs in saurischian dinosaurs like *Aerosteon*. Pleurocoels, pneumatic neural arches, and costal pneumatocoels closely match those in extant birds derived from diverticulae of the cervical air sacs. Posterior axial pneumaticity (posterior dorsal, sacral, and caudal vertebrae) occurs sporadically in nonavian saurischians and has been attributed either to cervical air sacs or to diverticulae extending directly from the lung and abdominal air sacs [23], [26], [27], [22], [31], [33], [35], [37]. In *Aerosteon* posterior axial pneumaticity has a similar form to that elsewhere among nonavian saurischians—a posterior extension without hiatus of the pneumatic pattern established in the anterior axial column.

In living birds, anterior and posterior axial pneumaticity develops during growth as diverticulae invade the axial skeleton from cervical air sacs, lung diverticulae, and caudal (abdominal) air sacs. These separate sources for axial pneumaticity can leave a pneumatic hiatus in the thoracic vertebrae, where the approaching diverticulae fail to fully anastomose. In *Gallus*, for example, there is an apneumatic hiatus in the thoracic vertebrae that may persist in the adult [20].

Wedel [26] pointed out that such a pneumatic hiatus has never been reported in saurischian dinosaurs that exhibit axial pneumaticity in presacral and sacral regions of the vertebral column. A hiatus might be expected to occur in young individuals, if axial pneumatization is derived from multiple sources as in living birds. Immature individuals of the ornithomimid *Sinornithomimus* as young as one year in age [56], however, show the same continuous and gradational pattern of presacral pleurocoels as occurs in subadults of the same species or adults of other ornithomimid genera [57].

Recently O'Connor proposed that axial pneumaticity in the abelisaurid theropod *Majungasaurus* can be used “to refine inferences related to pulmonary structure” [35: 159], because “it shows a reduction in the pneumaticity in the last two dorsal neural arches, with enhanced pneumaticity in sacral aches [31: 22]. Specifically, the “size and number of neural arch foramina” are reduced in dorsal vertebrae 12 and 13, whereas the same are “enhanced” in sacral vertebrae, indicating “two different sources of pneumatization” [33: 253]. The actual differences, however, were not described, and dorsal vertebrae 12 and 13 were not figured until recently [35: figs. 3, 12, 13].

Review of the posterior dorsal-to-sacral vertebral series in *Majungasaurus* suggests an equally plausible alternative explanation. The two posteriormost dorsal vertebrae have narrower, shorter transverse processes with single-headed ribs [35]. In preceding dorsal vertebrae, a pair of pneumatic fossae open on either side of the parapophysis (infraprezygapophyseal and infradiapophyseal fossae) as well as one farther posteriorly (infrapostzygapophyseal fossa). The loss of a distinct parapophyseal process eliminates the ridge of bone that divided the two anterior fossae, leaving a more broadly open area. Although not mentioned in the description, a distinct small funnel-shaped fossa is maintained in this area in dorsal 12 [[35: fig. 12C]], and a more broadly open fossa still remains in dorsal 13 [35: fig. 13A], representing the fusion of infraprezygapophyseal and infradiapophyseal fossae in both vertebrae. Counting the infrapostzygapophyseal fossa, two of the three fossae present in more anterior dorsal vertebrae appear in reduced form in these posteriormost dorsal vertebrae.

The mid sacral vertebrae are preserved, and each has two fossae visible on the ventral aspect of the diapophysis [35: fig. 13A]. Unlike any of the infradiapophyseal fossae in the presacral column, these are invaginated with a sharp-rimmed opening. The likely explanation for this is the increase surrounding bone from massive sacral attachments and fusion of adjacent neural arches. The pneumatic diverticulae thus are better surrounded by bone, compared to the posteriormost dorsal vertebrae with their reduced transverse processes.

It appears, in sum, that the “reduction” and “enhancement” of pneumatic fossae between posterior dorsal and sacral vertebrae in *Majungasaurus* is directly related to “reduction” and “enhancement” of vertebral structure. Regarding the reduced fossae in dorsal 12, O'Connor remarked, “Concomitant with the reduction in neural arch laminae, pneumatic foramina are only present immediately adjacent to the postzygapophyses” [35: 144]. This reduction of the size of the pneumatic fossae, nonetheless, was presented as prima facie evidence for separation of cervical and abdominal “focal centers” of pneumatic diverticulae [31: 22].

The situation in *Aerosteon* is instructive for the contrast that it provides across the same vertebral transition. In this case, pneumaticity appears to peak in the last dorsal, with a large pneumatic canal in the transverse process that is not present in sacral vertebrae (Figure 4C). The pleurocoels, in addition, develop a posterodorsally inclined partition in the posteriormost dorsal vertebrae that passes into the sacral series unchanged. The axial column of *Aerosteon* does not suggest a clean partitioning based on the number or size of pneumatic spaces, but rather a gradation in pleurocoel form that extends from the anterior cervical vertebrae through the distal caudal vertebrae. As in other saurischians, there is no pneumatic gap separating distinct zones.

O'Connor and Claessens [33: 253] have argued that the “general pattern of pneumaticity in *Majungasaurus* is expressed throughout Theropoda … indicating a consistent and widespread pattern of pneumatic invasion by caudally located air sacs in non-avian theropods.” Their cited tabulation of theropod vertebral pneumaticity, however, does not include any examples of axial pneumaticity partitioned by an apneumatic gap. The data from *Majungasaurus*, although well preserved and described [35], is not qualitatively different from information on saurischian vertebral pneumaticity that has been available for more than a century [58]–[60]. On this evidence alone, the source of posterior axial pneumaticity in nonavian saurischians is likely to remain controversial, even if posterior axial pneumaticity in most extant avians is derived from diverticulae of the lung and abdominal air sacs [26].

#### Gastral Pneumaticity

An extraordinary feature of *Aerosteon* is the pneumatic hollowing of several gastralia (Figures 10, 16E), the first such example in the nonavian dinosaur record. Pneumatopores that vary in shape from oval to more elongate enter the external (ventral) surface and open into pneumatic spaces that hollow sections of the shaft of two medial gastral elements. In one medial gastral element (fused to its opposite in the midline), a shallow external trough leads to an oval pneumatopore, which opens into a subcylindrical space within the shaft (Figure 10). The internal pneumatic spaces do not have partitions when prepared or viewed in computed tomographic section (Figure 16E).

The external (ventral) position of the pneumatopores suggests that the pneumatic diverticulae lay in superficial tracts outside the gastral cuirass. It seems unlikely that pneumatic diverticulae would penetrated the ventral thoracic wall to access external pneumatopores, when entering the gastralia directly from their internal (dorsal) surface would be much easier. A plausible explanation may be that these ventral pneumatic tracts are part of a subcutaneous system, which is present to varying degrees in birds and is composed of diverticulae from cervical, clavicular, and abdominal air sacs [1], [21], [22]. Subcutaneous diverticulae usually exit the thoracic cavity and extend under the skin to distant body surfaces. In the brown pelican (*Pelecanus occidentalis*), for example, diverticulae of the clavicular air sac exit the thoracic cavity dorsally and extend under the skin to reach the entire ventral surface of the thorax [61].

#### Appendicular Pneumaticity

Pneumaticity in pectoral and pelvic girdles may provide a more straightforward signpost for ventilatory air sacs, because these bones are pneumatized exclusively by ventilatory sacs in living birds. The median pneumatocoel in the furcula (wishbone) of *Aerosteon* is very similar to that in some living anseriforms (screamers, ducks, geese) (Figure 12B). Pelicaniforms (boobies, pelicans) also have a pneumatic furcula, but the bone is aerated via contralateral pneumatocoels on each clavicular ramus. Furcular pneumaticity in birds always involves diverticulae of the clavicular air sac, the most anterior of the cranial air sacs, which is located between the rami of the furcula (Figure 1, clas) [1], [16], [19], [22], [36].

The pneumatic openings on lateral and ventral aspects of the ilium in *Aerosteon* may have been aerated by the abdominal air sac. These include pneumatic tracts and pneumatopores on the lateral aspect of the blade (Figure 14A), pubic peduncle (Figure 14C), and ventral aspect of the brevis fossa (Figure 14D). They provide entry into the honeycomb (camellate) internal structure of the ilium (Figure 16F). Iliac pneumaticity in birds is developed exclusively by diverticulae of the abdominal air sac, which is located under the synsacrum as the most posterior of the caudal air sacs (Figure 1, aas). The openings on the lateral aspect of the iliac blade, given their external, superficial location, may be derived from subcutaneous diverticulae of the abdominal air sac [1], [16], [19], [21], [22], [36].

Some of the iliac pneumatic sculpting, nevertheless, may have been associated with posterior extension of the cervical air sac system. In particular, the pneumatopores on the medial aspect of the iliac blade (Figure 14B) and brevis fossa (Figure 14D) are adjacent to the sacral axial column and may have been pneumatized from the same source. As with posterior axial pneumaticity, the absence of extant analogs with similar pelvic and pneumatic structures weakens the case that all of the pneumatic features of the ilium originate exclusively from the abdominal air sac.

### Evolution of Avian Air Sacs and Lung Ventilation

#### Structural Phases

Tracking pneumatic patterns in the fossil record is complicated by the one-sided nature of outgroup comparison, which is restricted to birds among extant vertebrates, and the ambiguous meaning of the absence of a soft structure that only sometimes leaves an osteological imprint [55]. What we can sketch is the most probable minimal distribution of these soft structures and how they might have changed.

Pneumatic sculpting (fossae, pneumatocoels) of the cervical and anterior dorsal vertebrae in saurischians is widely understood as evidence of the presence of paraxial cervical air sacs, given the close correspondence with pneumatic structures observed in extant avians [22], [23], [31], [37] (Table 4). Fossils pertaining to a new basal theropod close to *Eoraptor* shows the presence of true pleurocoels in mid cervical vertebrae at the base of Theropoda [62], and it seems increasingly likely that a heterogeneously partitioned lung with air sacs were present in basal saurischian dinosaurs (Figure 17, bottom). The capacity of the cervical air sacs to invade centra to form invaginated pleurocoels may have evolved independently in sauropodomorphs (sauropods) and basal theropods and appears to have been lost several times within theropods.

**Table 4:** Osteological correlates of pneumatic features in the postcranial skeleton of nonavian dinosaurs based on observed pneumaticity in birds.

Soft Anatomy or Functional Capacity	Osteological Correlates
Pneumatic diverticulum	Pneumatic sculpting, preferably with a pneumatopore characterized by a smooth rim and an invaginated space with smooth walls and interconnected cells that are considerably larger than those in cancellous bone
Cervical air sac	Pneumatic invasion of cervical and dorsal (thoracic) vertebrae (centrum, neural arch) and ribs
Clavicular air sac	Pneumatic invasion of the furcula, coracoid, sternal ribs or humerus; furcular invasion preferably median on central body or parasagittal on epicleideal processes
Abdominal air sac	Pneumatic invasion of the pelvic girdle, preferably in areas removed from its contact with the sacrum (to avoid potential confusion with axial invasion by cervical air sacs)
Subcutaneous pneumaticity	Pneumatic invasion on an external bone surface at some distance from an air sac that would require superficial transmission
Costosternal pump	Ossification of sternal ribs and sternum; joints (synovial) between vertebral ribs, sternal ribs and sternum
Advanced costosternal pump	Concavoconvex joint (synovial) between coracoid and sternum; uncinate processes; dorsal (thoracic) column shortened
Flow-through ventilation (rigid lung)	Evidence of pneumatic invasion by at least one avian ventilatory air sac (clavicular, anterior or posterior thoracic, abdominal)
Uni- or bidirectional lung ventilation	None

*Phase I* of our model of the evolution of air sacs highlights the variable expression of the cervical air sac system in posterior portions of the axial column in theropods (Figure 17). Pneumatic sculpting of posterior dorsal, sacral and caudal vertebrae varies widely between taxa and sometimes between individuals of the same species. Such variation could parallel variation in the posterior extension of the cervical are sacs, or it may only indicate variation in the capacity of the posterior portion of the cervical air sac system for pneumatic sculpting of bone. Pneumatic fossae appear to be present on the centra along much of the axial column in the new basal theropod [62].

In *Phase II* additional air sacs are present in tetanuran theropods within the thorax, at least some with extrathoracic subcutaneous diverticulae (Figure 17). Reduction of skeletal weight, as a consequence, is no longer the only purpose served by air sacs. These additional air sacs include both cranial and caudal divisions of avian intrathoracic air sacs involved in lung ventilation (Table 4). The presence of compliant intrathoracic air sacs confirms deep-seated heterogeneous partitioning of the respiratory system in these theropods into mechanical areas involved in respiratory flow (ventilatory air sacs) from those involved in gas exchange (lung). The presence of ventilatory air sacs, in turn, suggests that the lung was rigid and most likely attached dorsally to the ribcage as in birds. Dorsal attachment of the lung has been inferred from stiffening of the dorsal vertebrae with extra (hyposphene-hypantrum) articulations, deeper dorsal rib attachments, and the presence of pleurocoels in dorsal vertebrae [33], [37], although the correlation between these osteological features and lung attachment seems poorly established. Although phase II theropods like *Aerosteon* display pneumatic sculpting consistent with cranial and caudal air sac divisions in birds, we do not claim they had unidirectional lung ventilation. Osteological correlates for directional lung ventilation, in our opinion, have yet to be established (Table 4).

*Aerosteon* currently provides the best evidence for intra- and extrathoracic air sacs and diverticulae, based on pneumatic invasion of the furcula, ilium and gastralia (Figure 16). As discussed above, we and others [26], [34] regard the relatively continuous pneumatic sculpting in the posterior axial column of nonavian dinosaurs as ambiguous evidence for abdominal air sacs [contra 22,31,33]. We anticipate that evidence from appendicular bones for these additional air sacs will be discovered in other species, and that this distribution may eventually encompass most theropods.

In *Phase III* a primitive costosternal pump is present, the evidence for which is best preserved in the basal ornithomimid *Pelecanimimus* [63], [64]. The holotype and only known specimen (LH 7777) has a pair of elongate ossified sternal plates and several pairs of ossified sternal ribs. The coracoid remains hook-shaped as in other ornithomimids and lacks the square distal end that characterizes the coracoid in maniraptorans. Unlike maniraptorans, the coracoid does not contact the sternum, and there is no trace of ossified uncinate processes.

Complete, articulated skeletons of other ornithomimids show no trace of sternal ossifications [56], [57], except for a pair of rod-shaped “xiphisternal” elements in *Struthiomimus* [65]. Sternal ossifications are also absent in another toothed basal ornithomimid that preserves most of the skeleton [66]. The rare ossification of sternal structures in ornithomimids, when their existence in cartilage was correctly inferred [65], highlights the problematic nature of trying to establish accurate distributions for characters related to respiratory function. Retention of dorsal ribs posterior to those attached to the sternum as well as a full gastral cuirass with mobile median articulations suggests that, in spite of the presence of a large ossified or cartilaginous sternum, the costal and gastral skeleton continued to play a role in aspiration breathing.

In *Phase IV* the costosternal pump is more birdlike still (Figure 17), as seen in the basal oviraptorosaur *Caudipteryx* [67] and in several dromaeosaurids [68]–[70]. The ribcage is proportionately shorter anteroposteriorly and is crossed by a series of ossified uncinate processes, which have been linked to respiratory mechanics in living birds [71]. The large paired sternal plate articulates with ossifed sternal ribs laterally and has a thickened anterior end marked by a groove to receive the ventral edge of the coracoid [68], [70]. Synovial joints thus appear to be well formed between the sternum and the sternal ribs and coracoid. Although the sternal ribs have not been shown to have transversely expanded distal ends for articulation with the sternum as occurs in birds [34], it is difficult to deny that the ribcage and pectoral girdle are mechanically united in a manner very similar to that in birds [70]. The primary respiratory role played by uncinate processes in living birds [71] suggests that the derived form of the ribcage and pectoral girdle in phase IV theropods served a ventilatory role as a costosternal pump. Again, whether that ventilation was uni- or bidirectional we regard as impossible to ascertain from current evidence.

#### Lung Ventilation, Air Sacs

Two general models have been proposed for lung ventilation in nonavian dinosaurs. The first infers the presence of compliant lungs with crocodile-like diaphragmatic ventilation, based in part on stained areas in two theropod skeletons purported to represent a diaphragm separating thoracic and abdominal cavities [72], [73]. The stains and their interpretations have been contested, and the evidence for their arguments refuted by several authors [22], [25]–[27], [37]. A second model infers avianlike flow-through lung ventilation with a rigid dorsally-attached lung and compliant air sacs. This hypothesis is based mainly on the morphology of the ribcage and on pneumatic sculpting in the axial column attributable to air sacs [24], [33], [64], [70]. Although more plausible, the second hypothesis actually consists of a collection of inferences about (1) pulmonary morphology and function and (2) the mechanics of aspiration respiration in nonavian dinosaurs. The osteological or logical correlates needed to support some of these inferences have been poorly articulated, which may explain the wide range of opinions on when intrathoracic air sacs like those in birds first evolved and how these relate to ventilatory patterns. We assemble the osteological correlates we used as a guide in assembling the four-phase model outlined above (Table 4). Below we first consider inferences about air sacs and later those involving the ribcage and gastral cuirass.

Some of the initial reconstructions of the pulmonary condition in nonavian dinosaurs were characterized as the “stepwise transformation of a crocodilian-like lung into an avian airsac system” [74: 132]. Such a transformational approach presumes that the crocodilian lung is primitive for archosaurs, which may not be the case. For nonavian dinosaurs, avians are the first and only outgroup for many of the pneumatic structures of interest, and so identifying osteological correlates and their probable functional associations is the best available approach [55].

Perry [75: 58] outlined a “dinosaur grade” pulmonary system that involved a dorsally attached lung, avian-like air sacs, bi-directional air flow, and costal-driven aspiration. Later [74], [76: 134] it was postulated that the “dynamic gastralia” of theropods suggests there were abdominal air sacs, which in turn are “crucial for unidirectional flow in the paleopulmo of birds.” On this basis, anterior and posterior air “chambers” were inferred to have evolved at the base of Dinosauria and “true airsacs” to have evolved at the base of Theropoda [76: fig. 1].

Based on the osteological correlates we have assembled (Table 4), we would argue, first, that until we can show evidence of the presence of at least one avian ventilatory air sac (besides the non-ventilatory cervical air sac), it is problematic to infer the presence of flow-through ventilation or a rigid, dorsally-attached lung. Second, we know of no osteological correlates in the gastral cuirass that would justify the inference of abdominal air sacs. Potential kinesis of the gastral cuirass and an accessory role in aspiration breathing potentially characterizes many amniotes besides nonavian dinosaurs [77]–[79]. The absence of gastralia in crown birds or in any extant bipeds also hinders functional inferences. And third, it is not well established that abdominal air sacs were either first to evolve or are functionally critical to unidirectional ventilation.

Experimental studies on ventilation in birds have shown slight changes in the orientation of air sacs relative to the connecting air passages can convert bidirectional to unidirectional flow in a poorly valved respiratory tract [2], [77], [78]. The slight pressure differences driving the particular ventilatory pattern have no known osteological correlates. A heterogeneous pulmonary system composed of a rigid lung and single air sac effectively separates the exchanger and pump, respectively, and would suffice to drive bidirectional or even unidirectional lung ventilation, given appropriate valving. The cranial-caudal air sac divisions and two-breath respiratory cycle of extant avians, thus, may have evolved from a simpler flow-through system.

Only the abdominal air sac in birds extends into the abdominal cavity, the other air sacs occupying more anterior spaces separated by septae [79]. Although the abdominal air sac may be more favorably located to play a ventilatory role [80], this has not been demonstrated in experimental systems or in living birds. All ventilatory air sacs are operative during lung ventilation in birds, with cranial and caudal divisions closely approximating each other in volume [2].

#### Lung Ventilation, Ribcage Function

Research on the gastral cuirass in archosaurs led to the suggestion that it may have functioned as an accessory aspiration pump in nonavian dinosaurs [81]–[83]. Although Claessens drew attention to the relationship between the gastral cuirass and abdominal air sacs, he concluded that “it appears impossible to ascertain exactly when lung diverticula stretching throughout the whole body cavity or unidirectional airflow originated” [83:102]. Later a “caudal origin model” for air sacs and flow-through lung ventilation (either uni- or bidirectional) was proposed [33: 255] based on (1) the presence of abdominal air sacs (inferred from posterior dorsal and sacral pneumaticity), (2) a dynamic gastral cuirass, and (3) vertebrocostal articulations in the posterior ribcage that allow greater excursion during aspiration (inferred from the more horizontal arrangement of posterior rib articulations). An independent study of rib morphology, in contrast, concluded that nonavian dinosaurs were characterized by an “anteriorly ventilated bellows lung” [84: 47].

In *Aerosteon* the dorsal rib articulations (especially the parapophysis) are quite large, and the diapophysis is always located dorsal to the parapophysis (Figures 7, 8) as in *Allosaurus* [47] and *Tyrannosaurus* [53]. An axis through these two rib articulations is canted anteroventrally from the vertical at about 5–10° in anterior dorsal vertebrae (Figure 7A, B) and increases to a maximum of about 25° in dorsal 11 (Figure 8A) before decreasing in the posteriormost dorsal vertebra (Figure 8B). In no part of the column does the does this costal articular axis approach the horizontal like that in abelisauroids [5], [35], [85] and some allosauroids [48].

Most or all of the gastral cuirass, in addition, is either fused or solidly articulated in the midline, which would prevent expansion of the cuirass as an accessory aspiration pump as proposed [33], [68], [81]–[83]. Although many nonavian dinosaurs may have employed costal- or gastral-driven aspiration, the condition in *Aerosteon* suggests that a more detailed comparative assessment of ribcage morphology and function is required. Currently we know of no osteological correlates in dorsal ribs or gastralia that would allow inference of either the presence of intrathoracic air sacs or a particular pattern of lung ventilation (Table 4) [34].

Avian lung ventilation is driven by muscles that expand and contract thoracic volume by deforming the ribcage and rocking a large bony sternum [4], [71]. Basal maniraptorans have many of the features associated with this ventilatory mechanism including a large ossified sternum, ossified sternal ribs, uncinate processes a deepened coracoid that contacts the sternum along a synovial hinge joint [68]–[70]. By contrast *Aerosteon* and the abelisaurid *Majungasaurus* lack these features. Does that mean that maniraptorans had evolved unidirectional lung ventilation? Or does it indicate only that the maniraptoran ribcage functioned in aspiration breathing more like that in avians? We do not know of any osteological correlates that are specifically tied to uni- or bidirectional lung ventilation (Table 4), which may explain the range of opinion as to how and when avian unidirectional lung ventilation first evolved.

#### Driving Factors

The factors driving the origin and evolution of the functional capacity of avian air sacs and lung ventilation remain poorly known and tested. We briefly mention these in the light of the new evidence from *Aerostoen*. Currently three factors have been proposed: (1) more efficient flow-through respiration, (2) locomotor balance in bipeds, and (3) thermoregulation. The first, perhaps the most common, is to construct intermediate morphologies between extant crocodilians and birds and presume that avian air sacs and unidirectional lung ventilation originated gradually for the respiratory functions they now perform [74]–[76], [86]. Given that many dinosaurs probably had elevated metabolic rates, more efficient lung ventilation may have been required [72], [73], although others have argued that gas exchange was not a limiting factor [87]. This hypothesis is difficult to test without better data from the fossil record and reliable osteological correlates for soft tissues and specific functions.

The second is that air sacs may have arisen originally for locomotory control in bipeds as a means to lower the center of mass and reduce rotational inertia [88], [89]. Evidence of air sacs, however, does not seem strongly correlated with the advent of bipedalism among dinosaurian precursors and its maintenance among descendants. Besides bipedal theropods, four-legged sauropods provide the best evidence among nonavian saurischian dinosaurs of air sacs, which do not seem to have been present in the broad array of bipedal ornithischians. Bipedalism, moreover, may have arisen as an upright posture that reduced mechanical conflict between locomotion and breathing [90], [91] rather than as a catalyst for air sacs.

Finally thermoregulatory control may have played a role in the origin of air sacs [26]. Living birds lack sweat glands, which in mammals reduce excess body heat by evaporative loss from highly vascularized surface tissues. Could air sacs or their intermuscular and subcutaneous diverticulae initially have played a similar role in heat transfer? *Aerosteon* may have had a fairly extensive system of subcutaneous air sacs enveloping the thoracic cavity. The fossil evidence for intrathoracic air sacs now closely overlaps that for feathers, which had evolved in coelurosaurian theropods most likely for heat retention [92]. Air sacs may have initially been employed as an antagonist to feathers in theropod thermoregulation. Although this hypothesis has been criticized for lack of empirical evidence in living birds [22], air sacs have been implicated in avian heat transfer and/or evaporative heat loss [93]–[95], and *Aerosteon* and many other theropods had a body weight more than an order of magnitude greater than that for any living bird. A thermoregulatory role for the early evolution of air sacs in nonavian dinosaurs should not be ruled out without further evidence from nonvolant ratites.

In sum, although we may never be able to sort out the most important factors behind the origin and evolution of the unique avian pulmonary system, discoveries such as *Aerosteon* provide clues that help to constrain the timing and circumstances when many of the fundamental features of avian respiration arose.

### Conclusions

We present a four-phase model for the evolution of avian air sacs and costosternal-driven lung ventilation based on the known fossil record of theropod dinosaurs and osteological correlates in extant birds:

1. Phase I—Elaboration of paraxial cervical air sacs in basal theropods no later than the earliest Late Triassic.
2. Phase II—Differentiation of avian ventilatory air sacs, including both cranial (clavicular air sac) and caudal (abdominal air sac) divisions, in basal tetanurans during the Jurassic. A heterogeneous respiratory tract with compliant air sacs, in turn, suggests the presence of rigid, dorsally attached lungs with flow-through ventilation.
3. Phase III—Evolution of a primitive costosternal pump in maniraptoriform theropods before the close of the Jurassic.
4. Phase IV—Evolution of an advanced costosternal pump in maniraptoran theropods before the close of the Jurassic.
  In addition, we conclude:
5. The advent of avian unidirectional lung ventilation is not possible to pinpoint, as there are no known osteological correlates for uni- or bidirectional lung ventilation.
6. The origin and evolution of avian air sacs may have been driven by one or more of the following three factors: flow-through lung ventilation, locomotory balance, and/or thermal regulation.
